# Chloroquine for COVID-19: rationale, facts, hopes

**DOI:** 10.1186/s13054-020-02932-4

**Published:** 2020-05-08

**Authors:** Andrea Cortegiani, Mariachiara Ippolito, Giulia Ingoglia, Sharon Einav

**Affiliations:** 1 Department of Surgical, Oncological and Oral Science (Di.Chir.On.S.), Section of Anaesthesia, Analgesia, Intensive Care and Emergency, Policlinico Paolo Giaccone, University of Palermo, Palermo, Italy; 2 Intensive Care Unit of the Shaare Zedek Medical Centre and Hebrew University Faculty of Medicine, Jerusalem, Israel

**Keywords:** COVID-19, Chloroquine, SARS-CoV-2, Pneumonia, Coronavirus

The tragedy of the pandemic coronavirus disease 2019 (COVID-19) led to a desperate search for effective treatments. Chloroquine (CQ), an aminoquinoline used for many years for the prophylaxis and therapy of malaria and autoimmune diseases, has been put forward as a treatment option.

The fact that CQ is not patented and has been in clinical use for years is a major advantage. CQ has been shown to have antiviral effects in SARS, MERS, Ebola, and HIV infections, but without data showing clinical effectiveness [1, 2]. Does the current level of evidence suffice for prescribing CQ for COVID-19?

## Rationale

Not every exposure to SARS-CoV-2 correlates with infection, since its infectivity also depends on environmental and host characteristics. Emerging evidence suggests the progression of COVID-19 is characterized by two possibly overlapping phases. During the early phase, host viral load is high, and even in the presence of pneumonia, systemic damage is limited. In the later phase, viral load decreases, but elevated cytokine levels and a hyper-inflammatory response are accompanied by damage to other organs [3].

**Fig. 1:**
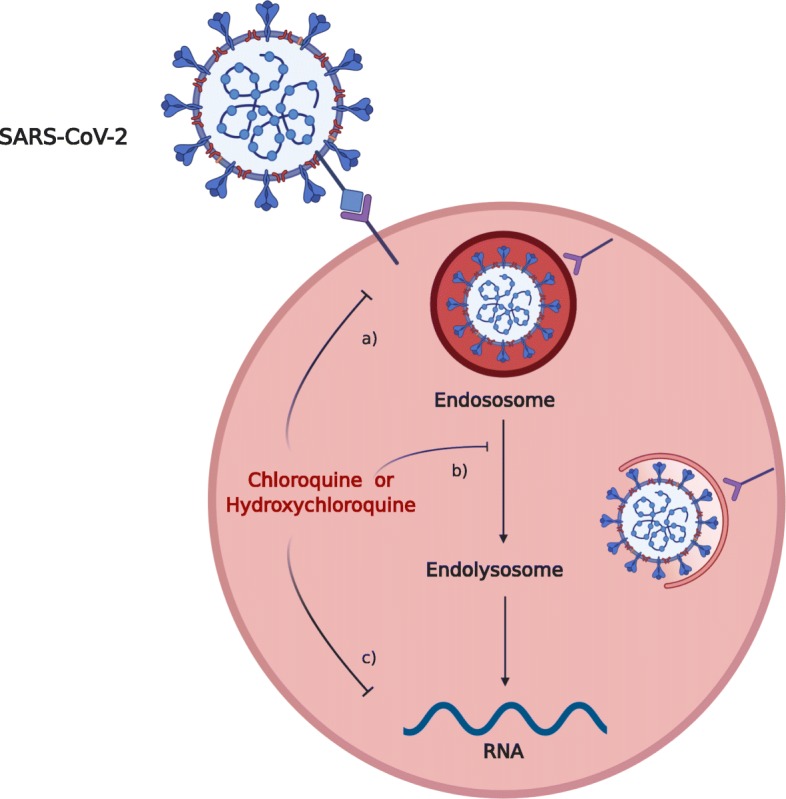
Proposed mechanisms for chloroquine and hydroxychloroquine effectiveness on SARS-CoV-2 infection. The mechanisms proposed as responsible for the effects of chloroquine and hydroxychloroquine: (a) the drugs interfere with the terminal glycosylation of cellular receptor ACE-2, thus hampering virus-receptor binding; (b) the drugs increase the pH of acidic cellular organelles, hindering endocytosis at intermediate stages with negative effects on virion transport and potentially altering post-translational modification of newly synthesized viral proteins; and (c) the drugs may contrast the process of virion assembly and viral protein synthesis

Several mechanisms have been proposed to assume that CQ or hydroxychloroquine (HCQ) may be effective against SARS-CoV-2 (Fig. 1) [1, 2]:

- Cell models of SARS-CoV-1 infection treated with CQ show interference with the glycosylation of ACE-2 receptors, proposed as the site of SARS-CoV-2 cell binding.
- CQ/HCQ increases the pH of acidic cellular organelles, hindering the intermediate stages of endocytosis and virion transport and post-translational modification of newly synthesized viral proteins.
- CQ/HCQ can counter the process of virion assembly and viral protein synthesis.

CQ also downregulates cytokine (e.g., TNF-α) production by monocyte-macrophages.

Although these effects suggest CQ/HCQ may affect infectivity and replication of SARS-CoV-2, previous experience with drugs attempting to modulate virus infection and the autoimmune septic response at the cellular level shows that bench and bedside results do not always correlate.

## Facts

The first description of CQ in SARS-CoV-2 infection was an in vitro study evaluating the effect of five antiviral drugs on infected Vero E6 cells. CQ showed effectivity at clinically acceptable concentrations [EC90 6.90 μM] [4]. The authors provided no information about the CQ formulation used. HCQ sulfate manifested similar effects with a significantly lower EC50 [5]. Another preclinical study confirmed the superiority of HCQ sulfate over CQ phosphate, by showing lower EC50 and higher inhibition rates [6]. Based on pharmacokinetic models, these authors proposed that a loading dose of 400 mg BID HCQ sulfate, followed by 200 mg BID, would maintain effective drug concentration in lung tissue.

One observational study reported data on treatment with HCQ sulfate in patients infected with SARS-CoV-2 in France. The authors compared nasopharyngeal swab viral loads over 6 days in 20 patients treated with HCQ sulfate (200 mg TID for 10 days) and in 16 patients who refused or had contraindication to treatment [7]. The clinical severity of the patients ranged between asymptomatic and pneumonia, but none was critical. On the sixth day, less patients had a detectable viral load in the HCQ group and the effect seemed more evident in the six patients who received azithromycin in addition to HCQ. The small number of participants (*n* = 36), lack of control for confounders, brief follow-up, and substantial loss to follow-up among those treated (23%, 6/26) limit the validity of these findings. The authors also did not use an intention-to-treat analysis, although they did declare the reasons for patient dropouts. Finally, the indications for combined HCQ-azithromycin treatment were not described. On April 3, the International Society of Antimicrobial Chemotherapy (ISAC) declared that “The ISAC Board believes the article does not meet the Society’s expected standard, especially relating to the lack of better explanations of the inclusion criteria and the triage of patients to ensure patient safety.” highlighting that “the need for fast release of new data should not reduce the quality of scientific scrutiny” [8].

And what about the safety profile of CQ/HCQ? This too derives from their long-term use in other clinical settings. Common side effects are QT prolongation, hypoglycemia, mental status changes, and retinopathy. Monitoring of heart rate and the QT interval, glucose levels, hepatic and renal function, and clinical screening for mental and visual disturbances are therefore recommended in patients receiving these drugs [9].

## Hopes

Despite lack of proof, guidelines of several countries propose various formulations of CQ for consideration in the treatment of patients with COVID-19, often referring to locally available formulations. The base form of CQ/HCQ is dissimilar from phosphate or sulfate formulations; 300 mg of CQ base corresponds to 500 mg of CQ phosphate, while 155 mg of HCQ base corresponds to 200 mg of HCQ sulfate. Chinese guidelines proposed CQ phosphate 500 mg BID for 7 days [10]. The Italian Society of Infectious Diseases recommends 500 mg CQ phosphate or 200 mg HCQ sulfate BID for 10 days regardless of severity, but recommends against prophylactic use [11]. The COVID-19 Surviving Sepsis Campaign guidelines made no recommendation on the use of CQ/HCQ in critically ill COVID-19 patients due to insufficient evidence [12].

A large number of ongoing trials [2] are an indicator of an idea gone rampant, not an indicator of effectiveness. Only a rigorous randomized controlled trial (RCT) can provide reliable and generalizable data regarding clinical effects of CQ/HCQ in COVID-19 [13]. The WHO recently published a global call to join an adaptive RCT of treatment in patients with COVID-19 [14]. The trial aims to establish the efficacy and safety of antiviral treatments on mortality in this population, and CQ is one of the four treatment arms.

In the frenzy to save patients, the story of CQ may be repeated: description of in vitro activity against SARS-CoV-2 of an “old drug” (as the recent case of the anti-parasitic ivermectin [15]), drawing huge media attention and incentivizing early publication of small studies in humans and empirical clinical use without quality data collection. Regardless of public pressure, clinicians should adhere to the national authorities’ regulations for prescribing off-label and experimental drugs, including CQ/HCQ.

## Data Availability

Not applicable

## Abbreviations

ACE: Angiotensin-converting enzyme
BID: Twice a day
COVID-19: Coronavirus disease 19
CQ: Chloroquine
EC50: Half-maximal effective concentration
EC90: 90% effective concentration
HCQ: Hydroxychloroquine
HIV: Human immunodeficiency virus
MERS: Middle East respiratory syndrome
RCT: Randomized controlled trial
SARS: Severe acute respiratory syndrome
TID: Three times a day
WHO: World Health Organization
